# Reproductive Outcomes in Young Women with Early-Stage Cervical Cancer Greater than 2 cm Undergoing Fertility-Sparing Treatment: A Systematic Review

**DOI:** 10.3390/medicina60040608

**Published:** 2024-04-06

**Authors:** Antonio D’Amato, Gaetano Riemma, Vittorio Agrifoglio, Vito Chiantera, Antonio Simone Laganà, Mislav Mikuš, Miriam Dellino, Annamaria Maglione, Raffaele Faioli, Andrea Giannini, Giuseppe Trojano, Andrea Etrusco

**Affiliations:** 1 Unit of Obstetrics and Gynecology, Department of Interdisciplinary Medicine (DIM), University of Bari “Aldo Moro”, Policlinico of Bari, Piazza Giulio Cesare 11, 70124 Bari, Italy; antoniodamato19@libero.it (A.D.); miriamdellino@hotmail.it (M.D.); 2 Department of Woman, Child and General and Specialized Surgery, University of Campania “Luigi Vanvitelli”, 80138 Naples, Italy; gaetano.riemma7@gmail.com; 3 Department of Health Promotion, Mother and Child Care, Internal Medicine and Medical Specialties (PROMISE), University of Palermo, 90127 Palermo, Italy; vittorio.agrifoglio@gmail.com (V.A.); vito.chiantera@unipa.it (V.C.); etruscoandrea@gmail.com (A.E.); 4 Unit of Obstetrics and Gynecology, “Paolo Giaccone” Hospital, 90127 Palermo, Italy; 5 Unit of Gynecologic Oncology, National Cancer Institute—IRCCS—Fondazione “G. Pascale”, 81031 Naples, Italy; 6 Department of Obstetrics and Gynecology, Clinical Hospital Center Zagreb, 10 000 Zagreb, Croatia; m.mikus19@gmail.com; 7 Gynecology and Obstetrics Unit, IRCCS “Casa del Sollievo della Sofferenza”, 71013 San Giovanni Rotondo, Italy; annamaria.maglione@libero.it (A.M.); raffaelefaioli@hotmail.it (R.F.); 8 Unit of Gynecology, “Sant’Andrea” Hospital, Department of Surgical and Medical Sciences and Translational Medicine, Sapienza University of Rome, 00189 Rome, Italy; andrea.giannini@uniroma1.it; 9 Department of Obstetrics and Gynecology, “Madonna delle Grazie” Hospital, 75100 Matera, Italy; giuseppe.trojano@asmbasilicata.it

**Keywords:** cervical cancer, early-stage, fertility-sparing treatment, trachelectomy, conization, neo-adjuvant chemotherapy

## Abstract

*Background and Objectives*: Despite advancements in detection and treatment, cervical cancer remains a significant health concern, particularly among young women of reproductive age. Limited data exists in the literature regarding fertility-sparing treatment (FST) of cervical cancers with tumor sizes greater than 2 cm. The objective of this systematic review was to evaluate the reproductive outcomes of women diagnosed with cervical cancer greater than 2 cm who underwent FST. *Materials and Methods*: A comprehensive search of the literature was carried out on the following databases: MEDLINE, EMBASE, Global Health, The Cochrane Library (Cochrane Database of Systematic Reviews, Cochrane Central Register of Controlled Trials, Cochrane Methodology Register), the Health Technology Assessment Database, and Web of Science. Only original studies (retrospective or prospective) that reported reproductive outcomes of patients with cervical cancer >2 cm were considered eligible for inclusion in this systematic review (CRD42024521964). Studies describing only the oncologic outcomes, involving FST for cervical cancers less than 2 cm in size, and case reports were excluded. *Results*: Seventeen papers that met the abovementioned inclusion criteria were included in the present systematic review. In total, 443 patients with a cervical cancer larger than 2 cm were included in this systematic review. Eighty pregnancies occurred, with 24 miscarriages and 54 live births. *Conclusions*: FST appears to be a viable option for women of childbearing age diagnosed with cervical cancer larger than 2 cm. However, careful consideration is advised in interpreting these encouraging results, as they are subject to limitations, such as variability in study designs and potential biases. In addition, reproductive outcomes should be further cross-referenced with oncologic outcomes to clarify the potential risk–benefit ratio. It is critical to conduct further research using standardized approaches and larger participant groups to strengthen the validity of the conclusions drawn.

## 1. Introduction

Cervical cancer, despite advancements in detection and treatment, remains a significant health concern, particularly among young women of reproductive age. Uterine cervix cancer ranks as the third most frequently occurring cancer among women under the age of 40 [1], and nearly 40% of cases involving early-stage cervical cancer occur in young women who wish to maintain their fertility and may consider fertility-preserving surgery as an option [2]. Furthermore, with the increasing average age of first pregnancy in Western countries, the risk of being diagnosed with cervical neoplasm before achieving reproductive goals has progressively risen [3]. Fertility-sparing treatment (FST) has emerged as a valuable option for preserving reproductive capacity in this population, especially for those with early-stage disease [4]. However, the optimal management of young women with early-stage cervical cancer, specifically those with tumor sizes greater than 2 cm undergoing FST, remains a subject of ongoing debate and investigation.

In the 2019 guidelines of the National Comprehensive Cancer Network (NCCN), a fertility-sparing approach continues to be recommended for IB1 tumors smaller than 2 cm in size [5]. Nonetheless, the guidelines emphasize that according to some authors, a 2 cm threshold could be utilized for vaginal radical trachelectomy (VRT), while a 4 cm threshold may be preferred for abdominal radical trachelectomy (ART) [6].

In this context, in recent decades, the occurrence and mortality rates of cervical cancer in affluent nations have declined due to the introduction of structured screening programs and advancements in diagnostic and prognostic methodologies [7]. Furthermore, considering the high 5-year survival rates exceeding 90% for early-stage cervical cancer, with a significant portion of these patients being in their reproductive years (up to 40%), the need for FST is evident [8].

In addition to exposure to HPV, the primary risk factor, various other factors contribute to the development of cervical cancer, including early initiation of sexual activity (before 16 years of age), multiple sexual partners, smoking, high parity, chronic oxidative stress, and socioeconomic disadvantage [9,10,11].

Understanding the reproductive outcomes in this specific subgroup is crucial for guiding clinical decision-making and improving patient counseling. Therefore, this systematic review aimed to comprehensively evaluate the existing literature on reproductive outcomes in young women with early-stage cervical cancer (>2 cm) undergoing FST, providing insights into the effectiveness and safety of these approaches in preserving fertility while ensuring optimal oncological and reproductive outcomes.

### Objectives

The aim of this systematic review was to evaluate the reproductive outcomes of women diagnosed with cervical cancer greater than 2 cm who underwent FST.

## 2. Materials and Methods

### 2.1. Eligibility Criteria

Only original studies (retrospective or prospective) that reported reproductive outcomes of patients with cervical cancer >2 <4 cm were considered eligible for inclusion in this systematic review. Studies describing only the oncologic outcomes, studies involving FST for cervical cancers less than 2 cm in size, and case report studies were excluded. Due to the limited amount of literature available on the subject, we did not exclude studies in which FST was proposed in selected cases, even for tumors larger than 4 cm.

### 2.2. Information Sources

This study was carried out according to the Preferred Reporting Items for Systematic Reviews and Meta-Analyses (PRISMA) guidelines [12], available through the Enhancing the Quality and Transparency of Health Research (EQUATOR) network, and the *Cochrane Handbook for Systematic Reviews* [13]. The study was registered with the international prospective register of systematic reviews (PROSPERO) under the registration number CRD42024521964.

MEDLINE, EMBASE, Global Health, the Cochrane Library (Cochrane Database of Systematic Reviews, Cochrane Central Register of Controlled Trials, Cochrane Methodology Register), Health Technology Assessment Database, Web of Science, and Research Register (ClinicalTrial.gov) were searched for studies describing patients who underwent FST for cervical cancer greater than 2 cm.

### 2.3. Search Strategy

The following medical subject heading (MeSH) and key search terms were used: “Uterine Cervical Neoplasm” (MeSH Unique ID: D002583) AND “2 cm” AND “Fertility sparing” (MeSH Unique ID: D059247) OR “Conservative treatment” (MeSH Unique ID: D000072700) AND “Trachelectomy” (MeSH Unique ID: D000069339) OR “Conization” (MeSH Unique ID: D019092) AND “Laparotomy” (MeSH Unique ID: D007813) OR “Laparoscopy” (MeSH Unique ID: D010535). We selected papers written in English from the inception of each database until 1 February 2024.

### 2.4. Study Selection

Titles and/or abstracts of studies retrieved using the search strategy were screened independently by 2 review authors (A.E. and A.S.L.) to identify studies that met the inclusion criteria.

The full texts of these potentially eligible articles were retrieved and independently assessed for eligibility by two other review team members (M.M. and A.D.). A manual search of the references of the included studies was also conducted to prevent the omission of pertinent research. 

Any disagreements between them over the eligibility of the articles were resolved through discussion with a third (external) collaborator. All the authors approved the final selection.

### 2.5. Data Extraction

Two authors (V.A. and V.C.) independently extracted data from articles about study features, characteristics of the included populations, FSTs, complications, and results/outcomes using a prepiloted standard form to ensure consistency. One author (M.D.) reviewed the entire data-extraction process.

### 2.6. Assessment of Risk of Bias

Two reviewers (A.M.M. and R.F.) independently assessed the risk of bias of studies included in this systematic review using a modified version of the Newcastle–Ottawa Scale (NOS) [14]. The quality of the studies was evaluated in five different domains: “study design and sample representativeness”, “sampling technique”, “description of the fertility-sparing treatment”, “quality of the population description”, and “incomplete outcome data” (Table S1). Any disagreements between the reviewers were resolved by a third reviewer (A.G.).

### 2.7. Outcome Measures and Data Synthesis

The primary objective of this study was to evaluate the reproductive outcomes of women with early-stage cervical cancer greater than 2 cm who underwent FST. Quantitative analysis was not possible due to data heterogeneity (including differences in the type of FST). We provided a descriptive synthesis of the results in separate sections based on the type of surgical approach employed for FST (trachelectomy, conization). The body of evidence on the effectiveness of FST for IA G2EC was assessed by two authors (A.E. and G.T.) using the Oxford Centre for Evidence-Based Medicine 2011 Levels of Evidence (OCEBM) [15].

## 3. Results

### 3.1. Study Selection

**Figure 1:**
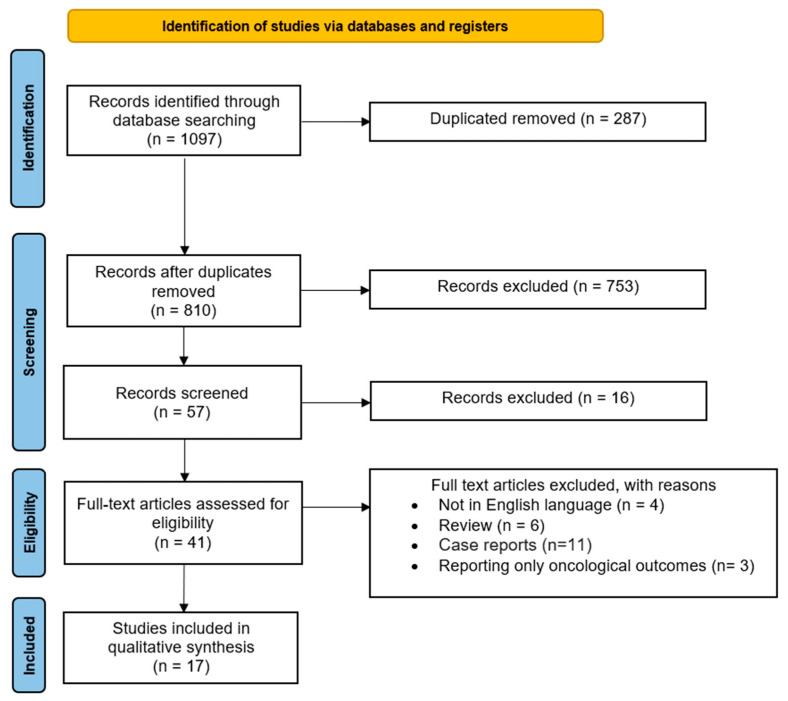
PRISMA flow diagram of the review.

The study selection process is displayed in Figure 1. After the evaluation of the full texts, 17 papers that met the abovementioned inclusion criteria [6,8,16,17,18,19,20,21,22,23,24,25,26,27,28,29,30] were included in the present systematic review.

### 3.2. Study Characteristics

The main characteristics of the included studies are summarized in Table 1. Four studies were prospective [16,17,26,29], and twelve were retrospective studies [6,8,18,19,20,21,22,23,25,27,28,30]. One study was a case series [24].

Of these, five studies were from China [6,16,18,19,22], two were from Germany [20,29], two from the Netherlands [28,30], one was from Italy [17], one from Hungary [21], one from France [23], one from Colombia [25], one from Czech Republic [26], one from Belgium [27], one from United States [8], and one from Canada [24].

### 3.3. Risk of Bias of Included Studies

Of the seventeen studies included, nine had a low risk of bias in three or more domains [8,16,17,19,20,21,22,23,25], and eight had a high risk of bias [6,18,24,26,27,28,29,30]. A detailed description of the risk of bias in each domain among the studies is reported in Table S2.

### 3.4. Synthesis of the Results

Among the included studies, 14 employed trachelectomy as the FST approach for cervical cancer greater than 2 cm [6,8,16,18,19,20,21,22,23,24,26,28,29,30], while in two studies conization was evaluated [17,27]. In one study, both approaches were employed [25]. Furthermore, NACT was administered before FST in 11 studies [17,20,22,23,24,25,26,27,28,29,30]. The range of tumor size and methodology used to establish it in the included studies are shown in Table 2. As previously mentioned, we discussed the results separately based on the type of FST approach used in the various included studies.

#### 3.4.1. Trachelectomy

Fifteen studies evaluated trachelectomy as an FST for patients affected by cervical tumors of a size exceeding 2 cm [6,8,16,18,19,20,21,22,23,24,25,26,28,29,30]. Of these, eight employed an abdominal approach [6,8,18,19,21,22,25,28], five used a vaginal approach [20,24,26,29,30], and one compared the effectiveness of both approaches [16]. Moreover, in one study, laparoscopic-assisted radical vaginal trachelectomy (LARVT) was utilized [23]. Additional information regarding the included studies can be found in Table 3.

Cao et al. [16] compared the surgical and fertility outcomes of 150 patients treated by either VRT or ART. Forty-eight patients (32%) were affected by cervical cancer greater than 2 cm: 24 in the VRT group (50%) and 24 in the ART group (50%). Twenty-four patients tried to conceive (50%) after successfully preserving fertility, and three pregnancies occurred, resulting in three live births (100%). Patients with tumor sizes greater than 2 cm exhibited significantly higher rates of recurrence (11.6% versus 2.4%, with a *p*-value less than 0.05) and lower rates of pregnancy (12.5% versus 32.1%, with a *p*-value of 0.094) compared to patients with tumor sizes less than 2 cm.

Deng et al. [18] assessed the surgical, oncologic, and fertility outcomes in 45 patients treated with sentinel lymph node biopsy (SNLB)-guided ART. Five pregnancies occurred, with four miscarriages (80%) and one full-term delivery (20%). Similar results were obtained by Guo et al. [19], Li et al. [6], and Lintner et al. [21]. In the series by Wethington et al. [8], no pregnancies occurred.

Lanowska et al. [20] evaluated the use of NACT before VRT for the FST of 20 patients. TP or TIP for two or three cycles was administered to all patients. Seven patients achieved pregnancy; of these, one miscarriage and one ectopic pregnancy occurred (28.6%). Eventually, four live births were obtained (57.1%), with another ongoing pregnancy reported by the authors. Comparable findings were achieved by Plante et al. [24], Robova et al. [26], Vercellino et al. [29], and Zusterzeel et al. [30].

Marchiolè et al. [23] evaluated the feasibility of LARVT after NACT with TP, TIP, or TEP in 19 patients. Three live births occurred in the series.

Lu et al. [22] employed an ART approach following NACT with bleomycin + cisplatin + mitomycin in seven patients. After surgery, four patients tried to conceive (57.1), and one live birth occurred (50%). Analogous outcomes were observed by Rendòn et al. [25], and Tesfai et al. [28].

**Quality of evidence:** The evidence regarding the safety, effectiveness, and reliability of FST employing trachelectomy for early-stage cervical cancers of a size greater than 2 cm was classified as evidence level 3.

#### 3.4.2. Conization

In three studies, conization was applied as the FST for cervical cancers of a size exceeding 2 cm [17,25,27]. Additional information regarding the included studies can be found in Table 4.

As previously mentioned, Rendòn et al. [25] employed both conization and trachelectomy for FST. In all the three included studies, NACT was administered before performing surgery.

De Vincenzo et al. [17] evaluated the use of NACT before cold-knife conization in 13 patients. The NACT regimen involved TP for three cycles in all patients. After surgery, three patients attempted to conceive (33.3%), and two pregnancies occurred, resulting in 2 live births (66.7%). Similar results were obtained by Rendòn et al. [25] and by Salihi et al. [27].

**Quality of evidence:** The evidence regarding the safety, effectiveness, and reliability of FST employing conization for early-stage cervical cancers of a size greater than 2 cm was classified as evidence level 3.

## 4. Discussion

The present qualitative analysis on reproductive outcomes in young women with early-stage cervical cancer (>2 cm) undergoing FST illuminates several critical points. Firstly, our findings underscore the importance of fertility preservation in this patient population, given the substantial proportion of women of reproductive age affected by cervical cancer. FST can offer promising reproductive outcomes, providing hope for future parenthood for these women. 

Since its inception by Prof Daniel Dargent in the late 1980s, VRT has undergone significant evolution. Dargent’s groundbreaking work revolutionized the management of early-stage cervical cancer by demonstrating the safe preservation of the uterine body and fertility potential in well-selected cases. Over time, this original procedure has evolved into various techniques, including ART, and more recently, laparoscopic and robotic radical trachelectomy (RRT) [3,20,31,32,33]. Furthermore, FST currently represents a feasible and well-established treatment option for other types of gynecological tumors [34,35,36]. With the implementation of new technologies in surgery [37] and the development and advancements in endoscopic surgery [38,39], minimally invasive treatment is increasingly evolving, becoming a reality.

An analysis of surveillance, epidemiology, and end results (SEER) data revealed that procedures preserving the uterus, such as cold-knife conization or trachelectomy, do not carry a significant risk of mortality when compared to hysterectomy [40]. In the same study, a tumor size greater than 2 cm represented a factor independently associated with worsened survival. In this context, the use of NACT for cervical cancers measuring 2–4 cm before FST is gaining significance, with the purpose of reducing tumor size, facilitating surgical removal, and mitigating adverse prognostic factors linked to suboptimal treatment response [41].

NACT, followed by surgery, enhances both overall survival and progression-free survival compared to surgery alone, resulting in a significant decrease in the risk of mortality [42]. Moreover, numerous studies have confirmed that NACT decreases the necessity for adjuvant radiotherapy while also reducing tumor size, lymph node involvement, and distant metastasis [26,43].

The status of lymph nodes plays a critical role as well, as a prognostic indicator, influencing the risk of recurrence and mortality [44]. However, there is currently no standardized approach for the timing of lymph node assessment compared to NACT, leaving this aspect uncertain [45,46]. Assessment of lymph nodes prior to neo-adjuvant chemotherapy seems the proper approach to better define patients with positive nodes, and, thus, poor prognosis, thereby excluding those most likely to require adjuvant treatment [20,27]. Furthermore, NACT administration before lymph node assessment may increase the pool of patients suitable for FST [47].

In total, the reproductive outcomes of 443 patients with a cervical cancer larger than 2 cm were evaluated in this systematic review. Among the included patients, 80 pregnancies occurred, with 24 miscarriages and 54 live births. The final pregnancy rate was 18.1%, with a miscarriage rate of 30% and a live birth rate of 67.5%. 

Regarding trachelectomy, it represents a feasible option for FST of early-stage cervical cancers, offering favorable oncological and reproductive outcomes, even though it represents a technique that needs to be tailored to each patient [48,49]. In the present systematic review, 77 pregnancies occurred in 430 patients after trachelectomy (17.9%), resulting in 50 live births (64.9%). 

On the other hand, with regard to conization, one study did not report disaggregated data about reproductive outcomes with conization or trachelectomy [25]; hence, it was possible to evaluate only the data pertaining to two studies [17,27]. Three pregnancies occurred among 23 patients (13%), all three resulting three live births (100%). Overall, the data currently available in the literature are too limited to make statements regarding the feasibility and reproductive outcomes of conization as an FST for cervical tumors larger than 2 cm [50]. 

However, we must also acknowledge the challenges and limitations associated with fertility-sparing approaches, including the risk of disease recurrence, the need for long-term follow-up to assess oncologic safety, and the rate of obstetric complications associated with surgical procedures. As evidence of this, the rate of infertility, premature rupture of the membranes, and premature delivery are significantly higher in patients who underwent trachelectomy [51,52]. In this systematic review, the rate of preterm delivery found after trachelectomy was up to 30%.

Regarding the pros and cons of the two procedures, trachelectomy offers the advantage of a more complete excision of the neoplasm but with the great downside of the impairment of fertility, mainly due to the excision of the parametrium and of the healthy cervical stroma extending beyond the tumor [53], the high miscarriage rate and preterm birth rate (approximately 30%, as found in this systematic review) [53,54], and adverse surgical outcomes in terms of urologic and neurologic morbidities [55]. On the other hand, conization is less invasive and associated with fewer complications but may not provide as high a chance of achieving a complete resection of the lesion, especially in cases of larger or more advanced tumors, such as cervical cancers greater than 2 cm. Ultimately, the choice between the two should depend also on individual patient factors and preferences. Moreover, the socioeconomic status of the patient is important both for cervical cancer prevention, access to fertility-sparing treatments, and for follow-up, as women with higher socioeconomic status will undergo more frequent check-ups, while females from disadvantaged socioeconomic backgrounds exhibit higher rates of incidence and mortality associated with cervical neoplasm [56]. In this regard, a recent population-based cohort study including 7736 young women with early-stage cervical, endometrial, or ovarian cancer undergoing FST demonstrated disparities in both clinical and sociodemographic factors affecting the utilization of FST among patients across different socioeconomic statuses [57].

### Limitations

Despite the valuable insights provided by this systematic review, several limitations should be acknowledged. Firstly, the lack of high-quality studies and the heterogeneity among the included studies may have introduced biases and limited the generalizability of our findings. Additionally, variations in study designs, patient populations, and treatment modalities across the included studies may have affected the reliability of the results.

## 5. Conclusions

In conclusion, our systematic review highlights the significance of FST for young women diagnosed with early-stage cervical cancer, particularly those with tumors larger than 2 cm. Despite the limited number of studies available, our analysis suggests that fertility-sparing approaches hold promise for preserving reproductive function in this population. 

However, the variability in treatment protocols and the lack of standardized guidelines underscore the need for further research to optimize patient selection criteria, refine therapeutic strategies, and enhance reproductive outcomes. Additionally, long-term follow-up studies are warranted to assess the oncologic safety and fertility preservation efficacy of these approaches. 

Overall, our findings emphasize the importance of personalized and multidisciplinary care in managing young women with early-stage cervical cancer, ensuring both oncologic control and preservation of fertility.

## Figures and Tables

**Table 1:** Characteristics of the included studies.

Author	Year	Type	Main Outcome	Country	Patient (n)	Cervical Cancer > 2 cm, n (% of total)	Age (Median)
Cao et al. [16]	2013	Prospective case-control	To compare the surgical and pathologic characteristics, the prognosis, and fertility outcomes of the patients treated by VRT or ART	China	150	48 (32)	30.0 (18–39)
De Vincenzo et al. [17]	2021	Prospective observational	To evaluate the feasibility of NACT followed by CKC in patients with 2018 FIGO stage IB2–IIA1 cervical cancer who desired to maintain fertility	Italy	13	9 (69.2)	29 (23–36)
Deng et al. [18]	2016	Retrospective observational	To evaluate the safety of SLNB-guided ART by observing surgical, oncologic, and fertility outcomes in patients who wished to preserve their fertility	China	45	45 (100)	28.5 (19–40)
Guo et al. [19]	2018	Retrospective observational	To compare the surgical and oncologic outcomes between ART and RH for stage IA2–IB1 cervical cancer	China	329	75 (22.8)	31 (18–42)
Lanowska et al. [20]	2013	Retrospective observational	To assess the oncologic and fertility outcomes of treatment in patients with cervical cancer of more than 2 cm seeking parenthood	Germany	20	20 (100)	32 (26–41)
Li et al. [6]	2013	Retrospective observational	To report the surgical and oncological safety of ART for selected patients with cervical cancer ≥2 cm in size	China	133	62 (46.6)	30.4 (20–44)
Lintner et al. [21]	2013	Retrospective observational	To report ART experience in patients with a cervical cancer more than 2 cm in diameter	Hungary	45	45 (100)	32 (24–43)
Lu et al. [22]	2014	Retrospective observational	To support the feasibility and safety of intra-arterial NACT followed by total laparoscopic radical trachelectomy in stage IB1 cervical cancer with a tumor larger than 2 cm	China	7	7 (100)	28 (22–35)
Marchiolè et al. [23]	2018	Retrospective observational	To assess the oncological and reproductive outcomes of patients with early-stage large cervical cancers (2–5 cm) undergoing FST	France	19	19 (100)	28.3 (21–37)
Plante et al. [24]	2006	Case series	To present the cases of 3 young women with bulky stage IB1 cervical cancer treated with NACT followed by laparoscopic pelvic node dissection and VRT	Canada	3	3 (100)	35 (26–36)
Rendòn et al. [25]	2020	Retrospective observational	To report on the oncological and obstetrical outcomes of NACT followed by FST in patients diagnosed with cervical cancer ≥2 cm	Colombia	25	25 (100)	27 (20–37)
Robova et al. [26]	2014	Prospective observational	To assess oncological and pregnancy outcomes after high-dose density NACT and FST in cervical cancer	Czech Republic	28	28 (100)	28.6 (15–34)
Salihi et al. [27]	2015	Retrospective observational	To discuss the cases of 11 patients with cervical carcinoma stage IB treated with NACT followed by large cone resection	Belgium	10	5 (50)	31.7 (25–36)
Tesfai et al. [28]	2015	Retrospective observational	To assess the feasibility, safety, oncological, and obstetric outcomes in patients with cervical tumors >2 cm treated with NACT in preparation for ART	Netherlands	19	19 (100)	28 (19–36)
Vercellino et al. [29]	2012	Prospective observational	To assess the results of treatment of women with stage I cervical cancer >2 cm in diameter seeking fertility preservation	Germany	18	6 (33.3)	31.3 (25–38)
Wethington et al. [8]	2016	Retrospective observational	To report the author’s trachelectomy experience with cervical tumors measuring 2–4 cm	United States	29	9 (31)	31 (22–40)
Zusterzeel et al. [30]	2020	Retrospective observational	To evaluate the oncological and fertility outcomes of treatment in patients receiving an FST consisting of NACT followed by VRT	Netherlands	18	14 (77.8)	29 (23–36)

VRT: vaginal radical trachelectomy; ART: abdominal radical trachelectomy; NACT: neo-adjuvant chemotherapy; CKC: cold-knife conization; SLNB: sentinel lymph node biopsy; RH: radical hysterectomy; FST: fertility-sparing treatment.

**Table 2:** Range of tumor size and methodology used for the assessment in the included studies.

Author	Tumor Size (cm)	Assessment
Cao et al. [16]	>2 <4	MRI or physical examination
De Vincenzo et al. [17]	>2 <4	MRI and physical examination
Deng et al. [18]	>2 <4	MRI
Guo et al. [19]	>2 <4	No data
Lanowska et al. [20]	2.1–5.0 (range)	MRI
Li et al. [6]	>2 <4	MRI, physical examination, or final pathology exam
Lintner et al. [21]	>2 <4 (55%) <2 (45%)	CT, PET-CT, or MRI
Lu et al. [22]	≥2.5 <4.0	MRI and physical examination
Marchiolè et al. [23]	≥2.9 <5.1	MRI and physical examination
Plante et al. [24]	>2 <4	MRI and physical examination
Rendòn et al. [25]	>2 <6	MRI (76%), CT (12%), physical examination (12%)
Robova et al. [26]	>2 (no data regarding maximum size)	MRI or TV-US
Salihi et al. [27]	>2 <5.2	MRI, CT or PET-CT
Tesfai et al. [28]	3.5–6.0 (range)	MRI
Vercellino et al. [29]	2.1–5.0 (range)	MRI, laparoscopic or hysteroscopic staging
Wethington et al. [8]	>2 <4	MRI, physical examination, or pathology exam
Zusterzeel et al. [30]	2.2–4.0 (range)	MRI and physical examination

MRI: magnetic resonance imaging; CT: computerized tomography; PET-CT: positron emission tomography-computerized tomography; TV-US: trans-vaginal ultrasonography.

**Table 3:** Reproductive outcomes of studies reporting FST with trachelectomy for early-stage cervical cancer greater than 2 cm.

	Cao et al. [16]	Deng et al. [18]	Guo et al. [19]	Lanowska et al. [20]	Li et al. [6]	Lintner et al. [21]	Lu et al. [22]	Marchiolè et al. [23]	Plante et al. [24]	Rendòn et al. [25]	Robova et al. [26]	Tesfai et al. [28]	Vercellino et al. [29]	Wethington et al. [8]	Zusterzeel et al. [30]
Patients, n	150	45	329	20	133	45	7	19	3	25	28	19	18	29	18
Year	2013	2016	2018	2013	2013	2013	2014	2018	2006	2020	2014	2015	2012	2016	2020
Cervical cancer greater than 2 cm, n (%)	48 (32)	45 (100)	75 (22.8)	20 (100)	62 (46.6)	45 (100)	7 (100)	19 (100)	3 (100)	25 (100)	28 (100)	19 (100)	6 (33.3)	9 (31)	14 (77.8)
Age, years (median)	30.0 (18–39)	28.5 (19–40)	31(18–42)	32 (26–41)	30.4 (20–44)	32 (24–43)	28 (22–35)	28.3 (21–37)	35 (26–36)	27 (20–37)	28.6 (15–34)	28 (19–36)	31.3 (25–38)	31 (22–40)	29 (23–36)
FST approach employed, n (%)	ART, 24 (50) VRT, 24 (50)	ART, 45 (100)	ART, 75 (100)	VRT, 20 (100)	ART, 55 (88.7)	ART, 45 (100)	ART, 7 (100)	LARVT	VRT, 3 (100)	ART, 20 (80)	VRT, 28 (100)	ART, 16 (84.2)	VRT, 6 (100)	ART or VRT, 9 (31)	VRT, 14 (100)
NACT, n (%)	0	0	0	20 (100)	0	0	7 (100)	19 (100)	3 (100)	25 (100)	28 (100)	19 (100)	6 (100)	0	14 (100)
NACT regimen, n (%)	-	-	-	TIP for 2 cycles, 15 (75) TIP for 3 cycles, 4 (20) TP for 2 cycles, 1 (5)	-	-	Bleomycin + cisplatin + mitomycin, 7 (100)	TIP for 4, 3 or 2 cycles, 11 (57.9) TP for 6, 4 or 3 cycles, 3 (15.8) TEP for 4 or 3 cycles, 5 (26.3)	TIP for 2 cycles, 3 (100)	TC, 8 (32) TP, 7 (28) TIP, 4 (16) Paclitaxel + cisplatin + 5- fluorouracil, 3 (12) 5- fluorouracil + ifosfamide + cisplatin, 2 (8) Vincristine + bleomycin + cisplatin, 1 (4)	Cisplatin + ifosfamide, 15 (53.6) Cisplatin + doxorubicin, 13 (46.4)	TP for 6 cycles, 11 (57.9) TP for 2 or 3 cycles, 8 (42.1)	TIP for 2 or 3 cycles, 6 (100)	-	TP for 2, 3 or 6 cycles, 14 (100)
Nulliparous, n (%)	112 (74.7)	20 (40.8)	n.d.	17 (85)	42 (67.7%)	n.d.	n.d.	n.d.	2 (66.7)	n.d.	26 (92.9)	n.d.	n.d.	24 (83)	14 (100)
Primiparous, n (%)	38 (25.3)	29 (59.2)	n.d.	3 (15)	20 (32.3%)	n.d.	n.d.	n.d.	1 (33.3)	n.d.	2 (7.1)	n.d.	n.d.	(17.2)	0
Attempted to conceive, n (%)	24 (50)	19 (42.2)	29 (38.7)	7 (35)	9 (16.3)	8 (25.8)	4 (57.1)	6 (31.6)	3	n.d.	16 (57.1)	15 (93.8)	6 (100)	2 (22.2)	7 (50)
Follow-up period, months (mean or median)	VRT, 34.4 ART, 20.6	37 (18–76)	75.5 (6–168)	23.1 ± 26.6	30.2	27	66 (12–90)	n.d.	n.d.	47 (13–133)	42 (5–103)	50 (3–144)	31.6	44 (1–90)	49.7 (11.4–110.8)
Pregnancies, n (%)	3 (12.5)	5 (26.3)	5 (17.2)	7 (100)	3 (33.3)	4 (50)	2 (28.6)	3 (50)	3 (100)	13 (56.5)	13 (81.3)	8 (53.3)	1 (16.7)	1 (50)	6 (85.7)
Spontaneous pregnancies, n (%)	n.d.	5 (100)	n.d.	7 (100)	n.d.	n.d.	n.d.	n.d.	n.d.	n.d.	10 (76.9)	8 (53.3)	n.d.	1 (100)	2 (33.3)
Assisted reproductive technologies, n (%)	n.d.	0	n.d.	0	n.d.	n.d.	n.d.	n.d.	n.d.	n.d.	3 (23.1)	0	n.d.	0	4 (66.7)
Miscarriages, n (%)	0	4 (80)	6 (75)	1 (14.3)	2 (66.7)	1 (25)	1 (50)	0	0	1 (7.7)	3 (23.1)	2 (25)	0	1 (100)	2 (33.3)
Ectopic pregnancies, n (%)	0	0	n.d.	1 (14.3)	0	0	0	0	0	0	0	0	0	0	0
Twin pregnancy, n (%)	0	0	0	0	0	0	0	0	0	0	0	0	0	0	0
Preterm delivery, n (%)	n.d.	0	n.d.	2 (28.6)	0	1 (25)	1 (50)	n.d.	1 (33.3)	7 (53.8)	3 (23.1)	0	0	0	0
Full-term delivery (%)	n.d.	1 (20)	n.d.	2 (42.8)	1 (33.3)	2 (50)	0	n.d.	2 (66.7)	4 (30.8)	5	6 (75)	1 (100)	0	3 (50)
Live births, n (%)	3 (12.5)	1	2 (25)	4 (57.1) 1 ongoing pregnancy	1 (33.3)	3 (75)	1 (50)	3 (100)	3 (100)	11 (84.6) 1 ongoing pregnancy	8 (61.5) 2 ongoing pregnancies	6 (75)	1 (100)	0	3 (50) 1 ongoing pregnancy
PR (%)	12.5	26.3	17.2	100	33.3	50	28.6	50	100	56.5	81.3	53.3	16.7	50	85.7
MR (%)	0	80	75	28.6	66.7	25	14.3	0	0	7.7	23.1	25	0	100	33.3
LBR (%)	100	20	25	57.1	33.3	75	14.3	100	100	84.6	61.5	75	100	0	50

FST: fertility-sparing treatment; VRT: vaginal radical trachelectomy; ART: abdominal radical trachelectomy; LARVT: laparoscopic-assisted radical vaginal trachelectomy; NACT: neo-adjuvant chemotherapy; TIP: paclitaxel, ifosfamide, and cisplatin; TEP: paclitaxel, epirubicin, and cisplatin; TP: paclitaxel and cisplatin; TC: paclitaxel and carboplatin; PR: pregnancy rate; MR: miscarriage rate; LBR: live birth rate.

**Table 4:** Reproductive outcomes of studies reporting FST with conization for early-stage cervical cancer greater than 2 cm.

	De Vincenzo et al. [17]	Rendòn et al. [25]	Salihi et al. [27]
Patients, n	13	25	10
Year	2021	2020	2015
Cervical cancer greater than 2 cm, n (%)	13 (100)	25 (100)	5 (50)
Age, years (median)	29 (23–36)	27 (20–37)	31.7 (25–36)
FST approach employed, n (%)	Cold-knife conization, 9 (69.2)	Conization, 5 (20)	Conization, 5 (50)
NACT, n (%)	13 (100)	25 (100)	5 (100)
NACT regimen, n (%)	TP for 3 cycles, 13 (100)	Carboplatin + paclitaxel, 8 (32) TP, 7 (28) TIP, 4 (16) Paclitaxel + cisplatin + 5- fluorouracil, 3 (12) 5- fluorouracil + ifosfamide + cisplatin, 2 (8) Vincristine + bleomycin + cisplatin, 1 (4)	TP or TP for 3 cycles, 1 (20) TC for 9 cycles, 4 (80)
Nulliparous, n (%)	12 (92.3)	n.d.	5 (100)
Primiparous, n (%)	1 (7.7)	n.d.	0
Attempted to conceive, n (%)	3 (33.3)	n.d.	5 (100)
Follow-up period, months (mean or median)	37 (18–76)	47 (13–133)	58 (13–122)
Pregnancies, n (%)	2 (66.7)	13 (56.5)	1 (20)
Spontaneous pregnancies, n (%)	2 (100)	n.d.	1 (100)
Assisted reproductive technologies, n (%)	0	n.d.	0
Miscarriages, n (%)	0	1 (7.7)	0
Ectopic pregnancies, n (%)	0	0	0
Twin pregnancy, n (%)	0	0	0
Preterm delivery, n (%)	1 (50)	7 (53.8)	0
Full-term delivery (%)	1 (50)	4 (30.8)	1 (100)
Live births, n (%)	2 (66.7)	11 (84.6) 1 ongoing pregnancy	1 (100)
PR (%)	66.7	56.5	20
MR (%)	0	7.7	0
LBR (%)	66.7	84.6	100

FST: fertility-sparing treatment; NACT: neo-adjuvant chemotherapy; TIP: paclitaxel, ifosfamide and cisplatin; TP: paclitaxel and cisplatin; TC: paclitaxel and carboplatin; PR: pregnancy rate; MR: miscarriage rate; LBR: live birth rate.

## Data Availability

Not applicable.

## Supplementary Materials

The following supporting information can be downloaded from https://www.mdpi.com/article/10.3390/medicina60040608/s1, Table S1: Modified Newcastle–Ottawa scale items, Table S2: Risk of bias assessment.
